# Prevalence of depression and anxiety among myasthenia gravis (MG) patients: A systematic review and meta‐analysis

**DOI:** 10.1002/brb3.2840

**Published:** 2022-12-10

**Authors:** Javad Nadali, Neda Ghavampour, Farzaneh Beiranvand, Mona Maleki Takhtegahi, Mohammad Eghbal Heidari, Shahin Salarvand, Tina Arabzadeh, Omid Narimani Charan

**Affiliations:** ^1^ School of Nursing and Midwifery Shahroud University of Medical Sciences Shahroud Iran; ^2^ School of Nursing and Midwifery Tehran University of Medical Sciences Tehran Iran; ^3^ Student's Scientific Research Center, School of Nursing and Midwifery Tehran University of Medical Sciences Tehran Iran; ^4^ Hepatitis Research Center, Nursing and midwifery faculty Lorestan University of Medical Sciences Lorestan Iran

**Keywords:** anxiety, depression, MG, systematic review

## Abstract

**Objective:**

Myasthenia gravis (MG) people experience adverse psychiatric outcomes, which may impact on their life and disturb their daily activity. Depression and anxiety are identified as significant psychiatric problems that MG people face. However, there is no sufficient epidemiological information about depression and anxiety‐based publication. Due to this limitation, the aim of this study was to review the prevalence of depression and anxiety in MG patients.

**Methods:**

Original and international databases were searched to find papers about the estimation of anxiety and depression. Random‐effects analysis was used for calculating the proportions of anxiety and depression. For estimating anxiety and depression based the severity, instruments, type of studies, and study regions, subgroup analysis was performed.

**Results:**

38 studies met inclusion criteria and entered study. The pooling of the prevalence of depression was found at 36%, (95% CI 28% to 45%). Also, prevalence of anxiety was found at 33%, (95% CI 25% to 42%). Prevalence of depression based on mild, moderate, and severe level was 27%, 14%, and 9%, respectively.

**Conclusions:**

Anxiety and depression are a major concern among MG individuals. The estimation of both anxiety and depression are high even when compared to other autoimmune diseases. It seems depression and anxiety are important issues and more attention needs to be paid to these psychiatric disorders.

## Graphical abstract

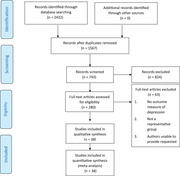

## 1 INTRODUCTION

Myasthenia gravis (MG) is identified as a prevalent autoimmune disease and the most common form of neuromuscular junction disease. The estimated prevalence of MG is about 20 individuals per 100,000 persons. Among gender, women are more susceptible rather than men (Cherukupally et al., 2020). It is predicted that MG affects 36,000 to 60,000 patients in the United States and more than 700,000 people worldwide annually. Age and sex are important factors in the incidence and prevalence of MG (Yamamoto et al., 2019). In MG, the transmission of nerve impulses to the muscles is disrupted. When there is no nerve–muscle connection, muscles are disrupted and cause neuromuscular disorders in these patients (Leopardi et al., 2021).

MG often occurs in adults and its common clinical manifestations include ocular, bulbar, or generalized weakness. Ocular complaints are the most common and are seen in 50% of cases. Due to the severity of symptoms such as weakness or fatigue, 30% of patients have respiratory problems and weakness and need mechanical ventilation (Hendricks et al., 2019).

Evidence showed that MG people experience psychological outcomes because of complication and recurrent symptoms. Mood disorders are the most common psychological outcomes among MG people (Kulaksizoglu, 2007; Paul et al., 2000; Qiu et al., 2010; Vitturi et al., 2021) Furthermore, about 20% of MG patients suffer from a psychiatric orpsychological disorder. Studies showed that prevalence of mental disorders and anxiety in people with MG are more than in the general population (Alanazy, 2019).

Furthermore, MG people experience different and complex treatments and invasive interventions which might impact their life and mental health. As the disease progresses, patients experience more disabilities and problems in their treatment that may reduce their quality of life and increase the incidence of mental disorders such as depression and anxiety (Yang et al., 2016). Numerous studies have reported that MG patients suffer from disability due to their symptoms, treatment, or complication (Alanazy, 2019; Qiu et al., 2010). Fatigue, drowsiness, anxiety, and depression are the most important complaints of patients (Alekseeva et al., 2019).

Psychological outcomes affect family, social, occupational, and personal aspects of patients (Parada et al., 2014). In addition, psychiatric and emotional disorders may lead to increased risk of mortality (Gavrilov et al., 2020; Kulaksizoglu, 2007).

According to studies about the prevalence of anxiety and depression in MG, the epidemiology of depression in MG varies from 6% to 76% (Kalbus et al., 2020; Sivadasan et al., 2019) and anxiety varies from 3% to 71% (Sivadasan et al., 2019; Ybarra et al., 2011). So, evidence showed that MG individuals are inclined to show high rate of depression and anxiety, and studies confirmed that anxiety and depression affect the mental health of MG people.

To sum up, many researchers have been evaluating the prevalence of depression and anxiety in MG patients and have had diverse results. For prevention and promotion of quality of life and treatment in MG patients, knowing the prevalence of depression and anxiety is necessary. Such information is essential for understanding the epidemiology of MG and is also helpful for developing strategies that help us to overcome the burdens caused by psychological manifestations of MG. Therefore, for achieving epidemiologic purposes, additional studies in different regions are necessary. According to the above statements and the absence of a systematic review or meta‐analysis of these issues, we conducted this study to determine the prevalence of depression and anxiety among MG patients all around the world.

## 2 MATERIALS AND METHODS

Preferred Reporting Items for Systematic Reviews and Meta‐analyses (PRISMA) were considered as guidelines for designing, performing, and writing study (Moher et al., 2009).

### 2.1 Search strategy

We fulfilled extensive search by searching the original databases of Web of Science, PUBMED, EMBASE, Ovid, and Scopus to find relevant and appropriate papers about estimate of anxiety and depression in MG patients. Search strategy was applied from 1960 to July 28, 2022 without any limitation on language and date. Manual searching in key journals for finding relevant articles was conducted after an initial search of databases and checking the reference list of included articles. Mesh terms, free text method, expert opinions as well as related articles and abstracts were checked to find the equivalent of search strategy terms. The terms used to search the databases were: (MG OR Myasthenia gravis) AND (depression OR Depressive disorder OR Cognitive disorder OR Anxiety OR mental disorder).

### 2.2 Study eligibility

Two authors (O.N. and M.E.H.) independently reviewed and retrieved articles to find out relevant study for including in the study according to titles and abstracts. Then, the full texts of papers were reviewed and studies were selected according to the inclusion criteria.

The inclusion criteria were: (1) observational studies (cohort, cross‐sectional), (2) papers which calculated or mentioned the proportions of depressive disorder or depression symptoms and anxiety, (3) an MG patient diagnosed by a physician, and 4) valid self‐reporting tools or structured diagnostic interviews for evaluating depression and anxiety. Case reports, interventional studies, magazine articles, case series, newspaper articles, or commentaries were considered as exclusion criteria. Articles that did not have complete information, had incomplete abstract or text, or did not provide sufficient and relevant data to estimate depression and anxiety were also excluded. Disagreements regarding the eligibility of studies were resolved by the third author (J,N).

### 2.3 Data extraction

The list of data extraction included publication year, study year, first author's name, design of study, study country, prevalence of depression and anxiety, mean age of participants, sample size, and score of Newcastle–Ottawa risk of bias. Articles that did not have the necessary information to calculate the prevalence of anxiety and depression were corresponded with the authors of the articles, and if they did not respond to the email more than three times, the studies were removed from the list of included studies.

### 2.4 Risk of bias (quality) assessment

Two authors investigated the quality assessment of articles. For evaluating the quality of studies, Newcastle–Ottawa risk of bias was used (Peterson et al., 2011).

### 2.5 Statistical analysis

STATA, version 12.0 (STATA Corporation, College Station, TX, USA) was used for data analysis. For each study, point estimates and 95% confidence intervals were calculated for the prevalence of depression and anxiety. The forest plot was also used to comprehensively represent selected studies based on consolidated estimated prevalence and 95% confidence interval. Meta regression was used to estimate the extent to which the measured covariates (year of release, sample size, and duration of disease) could explain the heterogeneity observed in prevalence estimates across studies. Publication bias was first assessed by visual inspection of the distribution of studies observed in a funnel design. Egger regression tracking (Vitturi et al., 2021) and Begg rank correlation test (Vitturi et al., 2021) were used to quantify the degree of bias shown in the funnel diagram.

## 3 RESULTS

### 3.1 Characteristics of included studies

**FIGURE 1:**
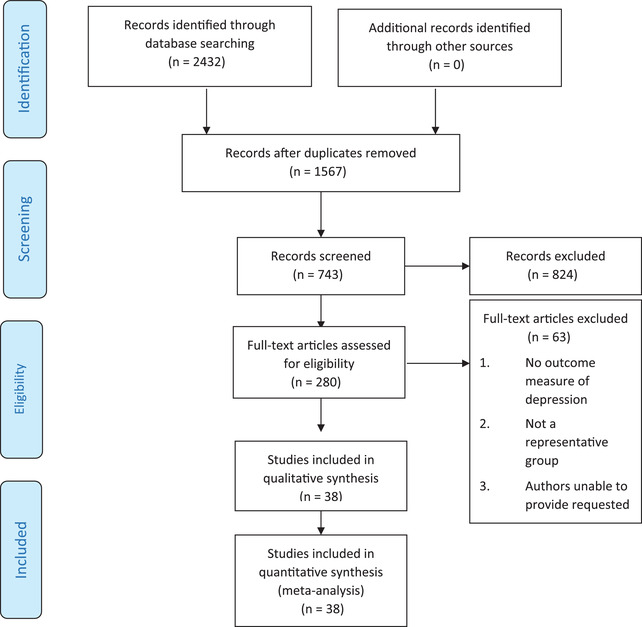
Four‐phase PRISMA flow diagram showing the number of studies identified, screened, eligible, and included in the systematic review and meta‐analysis

2432 citations were reviewed based on title and abstracts; 280 retrieved by assessing full text articles. So, 38 studies remained for final analysis (Figure 1). Table 1 outlines the characteristics of studies (2, 7–9, 12, 14–17, 20–48).

**TABLE 1:** Characteristics of the studies included in this meta‐analysis

First author	Year	Study location	Sample Size	Mean age	Study type	Instrument	*N* of total depression	*N* of Anxiety	*N* of anxiety/depression	*N* of mild depression	*N* of moderate depression	*N* of severe depression
**Vitturi**	2021	Brazil	49	18.1	Cohort	HADS			14			
**Chanchan**	2021	China	134	17.01	Cohort	HDRS	71	57				
**Kreis**	2021	Russian	73	15	Cohort	BDI	44			30	14	
**Kalbus**	2020	Ukraine	182	–	Cohort	BDI	139			47	18	36
**Yury**	2020	Switzerland	68	15.14	Cohort	BDI	44			30	12	2
**Bogdan**	2020	Canada	179	14.49	Cohort	BDI‐11	31					
**Annelise**	2020	Brazil	39	17.13	Cross‐sectional	BDI	13			5	8	3
**Kiana**	2020	Iran	62	13.3	Cross‐sectional	HDRS	40			21	9	10
**Asami**	2019	Japan	103	–	Cohort	DSM‐IV	10	6				
**Ajith**	2019	India	62	16.62	Cohort	–	4	2				
**Siddig**	2019	Sudan	33	–	Cross‐sectional	DSM‐IV	15					
**Hsuan‐Te**	2019	Taiwan	349	6.93	Cohort	–	22					
**Elizabeth**	2019	USA	242	13.4	Cohort	–	51					
**T. M. A**	2019	Switzerland	69	14.97	Cohort	BDI	14	18				
**Mohammed**	2019	Saudi Arabia	104	16	Cross‐sectional	PHQ‐9	27					
**Nida Fatma**	2017	Turkeys	19	13	Cross‐sectional	SCID‐1	4	4				
**Nayara, Braz**	2018	Brazil	80	14.17	Cross‐sectional	HADS	22	35		8	7	7
**Vanya**	2017	Bulgaria	97	–	Cohort	–	26					
**Jianyong**	2016	China	541	9.8	Cohort	–		179				
**Feray**	2016	Turkey	30	14.9	Cross‐sectional	BDI	18	17		8	7	3
**Sarah**	2016	Germany	200	17	Cross‐sectional	HADS	38	54				
**Soheir**	2016	Egypt	30	8.5	Cross‐sectional	SCID	13	15				
**Stefan**	2015	Australia	165	–	Cross‐sectional	–	26					
**C Freeman**	2014	South Africa	30	14.9	Cross‐sectional	BDI‐11	10	8				
**Syuichi**	2013	Japan	171	–	Cohort	–			3			
**Yasushi**	2011	Japan	287	17.1	Cross‐sectional	BDI	39					
**Fikret**	2013	Turkey	42	17.6	Cross‐sectional	BDI	17	4				
**Mariana**	2010	Brazil	41	13.4	Cross‐sectional	MINI	18	29				
**Yaroslav**	2010	Germany	37	20.2	Cross‐sectional				10			
**Qiu L**	2010	China	161	–	Cross‐sectional	HAM‐D	94	73				
**Di Blasi,**	2009	Italy	30	–	Cross‐sectional	BDI	30					
**Suzanne**	2007	USA	100	–	Cross‐sectional	CES‐D	42					
**Lundeen**	2004	USA	69	–	Cross‐sectional	–		38				
**Fisher**	2003	USA	45	–	Cross‐sectional	BDI	15			7	5	3
**Robert H**	2000	USA	29	13.78	Cross‐sectional	CMDI	5					
**T. M**.	2018	Russia	73	15	Cross‐sectional	BDI	44			30	14	
**Paradis CM**	2006	USA	35	19.05	Cross‐sectional	–		15				
**Hong**	2010	China	48	18.1	Cross‐sectional	HDRS	18			10	6	2

Abbrevations: BDI, Beck Depression Inventory; CES‐D, Center for Epidemiological Studies Depression; HDRS, Hamilton Depression Rating Scale; HADS, Hospital Anxiety and Depression Scale; PHQ, Patient Health Questionnaire; SCID‐1V, Structured Clinical Interview for DSM Disorders; DSM‐IV, Diagnostic and Statistical Manual of Mental Disorders; MINI, mini international neuropsychiatric interview; HAM‐D, Hamilton Depression Rating Scale; CMDI, Chicago Multiscale Depression Inventory.

In total, 38 studies involving 4108 individuals from 20 countries were included (Figure 1). Mean duration of disease was between 8.87 and 1.95 years. The mean age of participants was 47.04 (7.96) years. Dates of publication ranged from 2000 to 2021. Eleven studies were conducted in America, 15 were conducted in Asia, 10 were conducted in Europe, and 2 were conducted in African countries. Twenty‐four studies were cross‐sectional studies and 14 were cohort studies (Table 1). According to Myasthenia Gravis Foundation of America (MGFA), 835 individuals were in stages I and II, 365 patients in stage III, and 158 people with MG were in classification of IV.

The included studies applied different types of screening tools to assess anxiety. These tools included Beck Anxiety Inventory (BAI, four studies), Hospital Anxiety and Depression Scale (HADS, three studies), ADIS‐R, Diagnostic and Statistical Manual of Mental Disorders (DSM‐IV), GAD, HARS, STAI, and Structured Clinical Interview for DSM Disorders (SCID). For screening depression, used Beck Depression Inventory (BDI, 11 studies), HADS (3 studies), Hamilton Depression Rating Scale (HDRS, 3 studies), BDI‐II (2 studies), DSM‐IV (2 studies), CES‐D, PHQ‐9, SCID, HAMD, and Chicago Multiscale Depression Inventory (CMDI).

### 3.2 Results of the meta‐analysis

**FIGURE 2:**
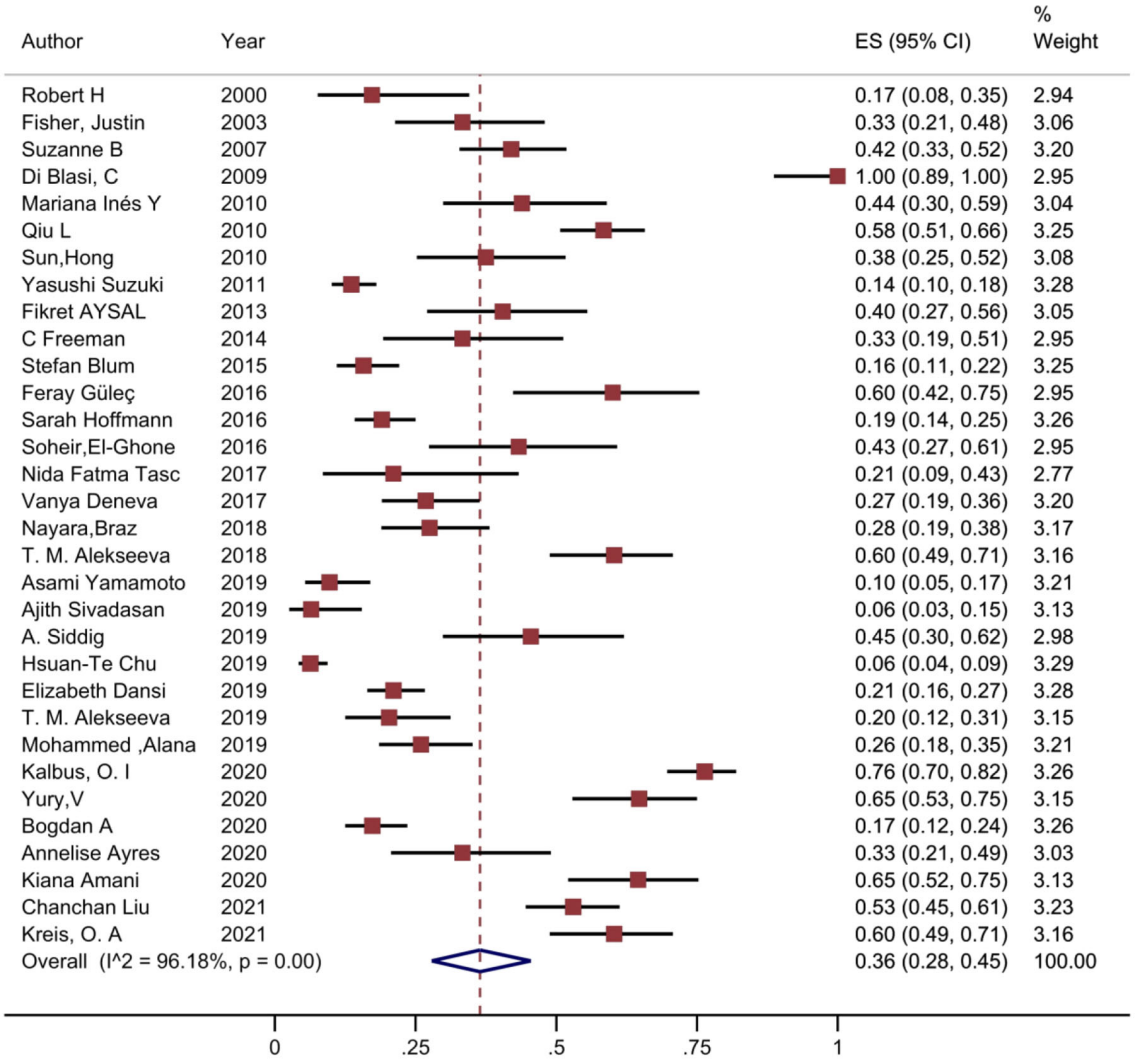
Forest plot of the prevalence of depression in MG patients. The 95% confidence interval for each study is shown in the form of horizontal lines around the central mean and midpoint of the dotted line represents the mean of the overall score and the lozenge shape shows the confidence interval of the prevalence of this disorder.

**FIGURE 3:**
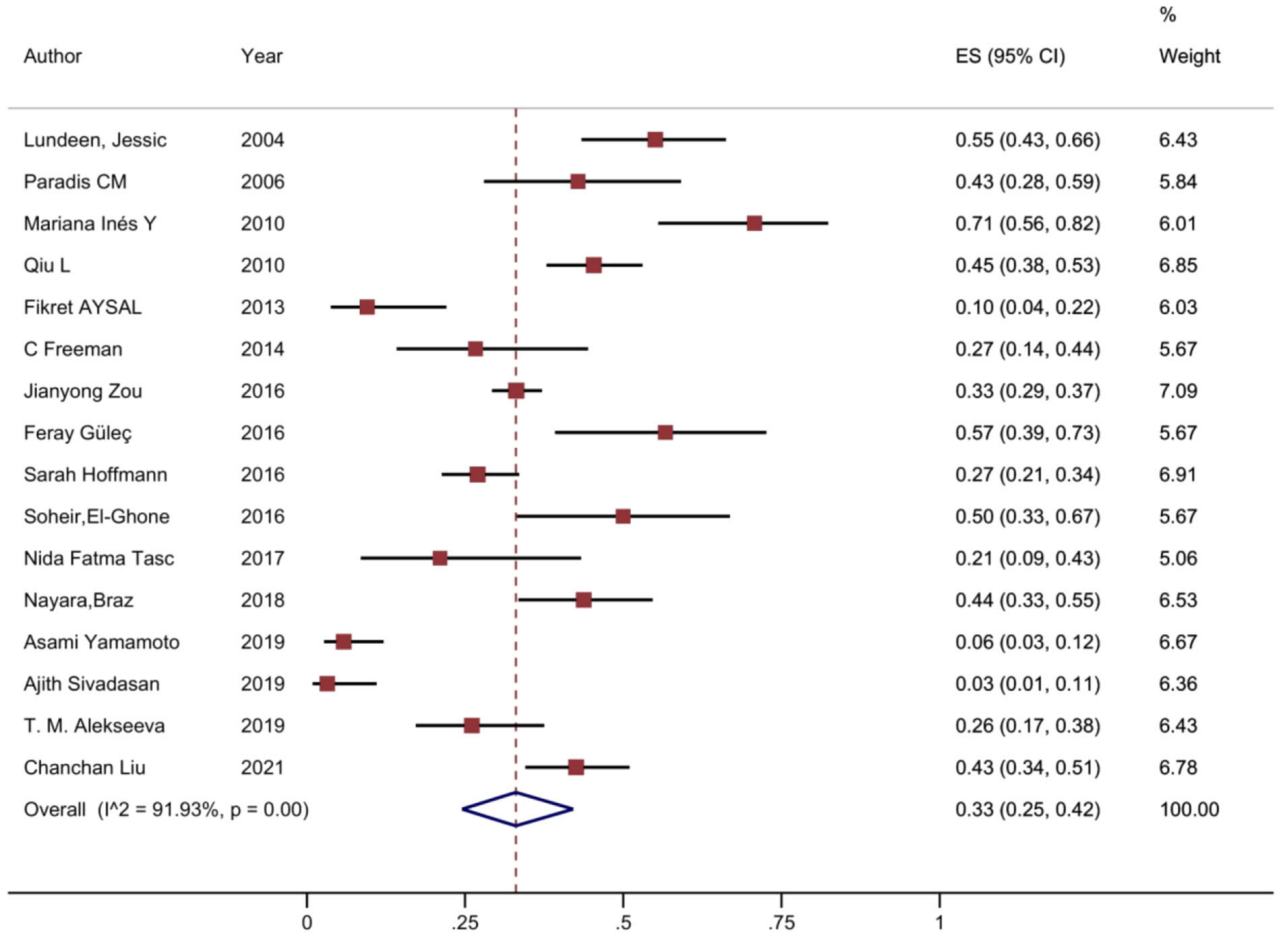
Forest plot of the prevalence of anxiety in MG patients. The 95% confidence interval for each study is shown in the form of horizontal lines around the central mean and midpoint of the dotted line represents the mean of the overall score and the lozenge shape shows the confidence interval of the prevalence of this disorder.

The pooled prevalence of depression reported was found at 36% (95% CI 28% to 45%) (Figure 2). Also, prevalence of anxiety was found at 33% (95% CI 25% to 42%) (Figure 3). Significant heterogeneity was found between studies in anxiety and depression. The test *I* ^2^ were 96.18% and 91.93% in depression and anxiety estimates, respectively. The prevalence of depression by the individual studies ranged from 1% to 76% and in anxiety ranged from 3% to 71%. Sensitivity analysis indicated that no individual study affected the overall prevalence estimate by more than 0.1%.

### 3.3 Subgroup analysis

For finding the source of heterogeneity and to characterize the range of depression and anxiety, subgroup analysis was performed based on the instrument, severity, continents, duration of disease, and type of studies. Prevalence of mild, moderate, and severe depression in MG people were 27%, 14%, and 9%, respectively. Also, further analysis based on severity of depression was conducted and the estimates of minor and major depression in MG were found at 31% and 26%, respectively. Based on the continent, the highest and lowest prevalence of depression was recorded in Europe (56%) and Asia (28%), respectively. Also, the prevalence of depression was 52% and 21% based on BDI and HADS, respectively (Table 2). In the cross‐sectional and cohort studies, the pooled prevalence of depression was 40% (95% CI: 30% to 50%) and 31% (95% CI: 16% to 48%), respectively (Table 2). Also, subgroup analysis showed the highest and lowest prevalence of anxiety was recorded in America (53%) and Asia (25%), respectively. In the cross‐sectional and cohort studies, the pooled prevalence of anxiety was 40% (95% CI: 31% to 51%) and 20% (95% CI: 8% to 36%), respectively.

**TABLE 2:** Results of subgroup analysis

Subgroup			Prevalence (%)	95% confidence interval	*I* ^2^, %	*p*
Type of tool						
Depression	BDI		52	0.33–0.72	97	< .001
	HDRS		52	0.39–0.65	–	
	BDI‐II		19	0.14–0.25	–	< .001
	HADS		21	0.17–0.26	–	< .001
	Self‐reported		14	0. 7–0.23		< .001
Severity of depression						
Depression	Mild	10 (Studies)	27	0.19–0.35	80	< .001
	Moderate	10 (Studies)	14	0.11–0.17	70	< .001
	Severe	8 (Studies)	9	0.05–0.15	68	< .001
Type of studies						
Depression						
	Cross sectional	27 (Studies)	40	0.30–0.50	93	< .001
	Cohort					
	11(Studies)	31	0.16–0.48	97	< 0.001	
Study regions						
Depression	America	8 (Studies)	29	0.21–0.36	78	< .001
	Europe	9 (Studies)	56	0.36–0.76	96	< .001
	Asia	13 (Studies)	28	0.17–0.41	96	< .001
	Africa	2 (Studies)	40	0.28‐0.52	–	< .001
Duration of disease						
Depression	< 2 years	8 (studies)	30	0.20–0.37	98	< .001
	> 2 years	11 (studies)	31	0.22–0.39	97	< .001
Type of studies						
Anxiety	Cross sectional	11 (Studies)	40	0.30–0.51	85	< .001
	Cohort	5 (Studies)	20	0.08–0.36	95	< .001
Study Regions						
Anxiety	America	4 (Studies)	53	0.41–0.65	68	< .001
	Europe	3 (Studies)	34	0.16–0.54	–	< .001
	Asia	8 (Studies)	25	0.14–0.37	94	< .001
	Africa	1 (Studies)	27	0.14–0.44	–	< .001
Duration of disease						
Anxiety	< 2 years	9 (studies)	32	0.23–0.40	98	< .001
	> 2 years	11 (studies)	29	0.19–0.35	96	< .001

### 3.4 Meta‐regression test

The prevalence of depression and anxiety in MG patients was not significantly related to mean age (*p* = .393), year of publication (*p* = .64), and duration of disease (*p* = .123).

### 3.5 Publication bias

**FIGURE 4:**
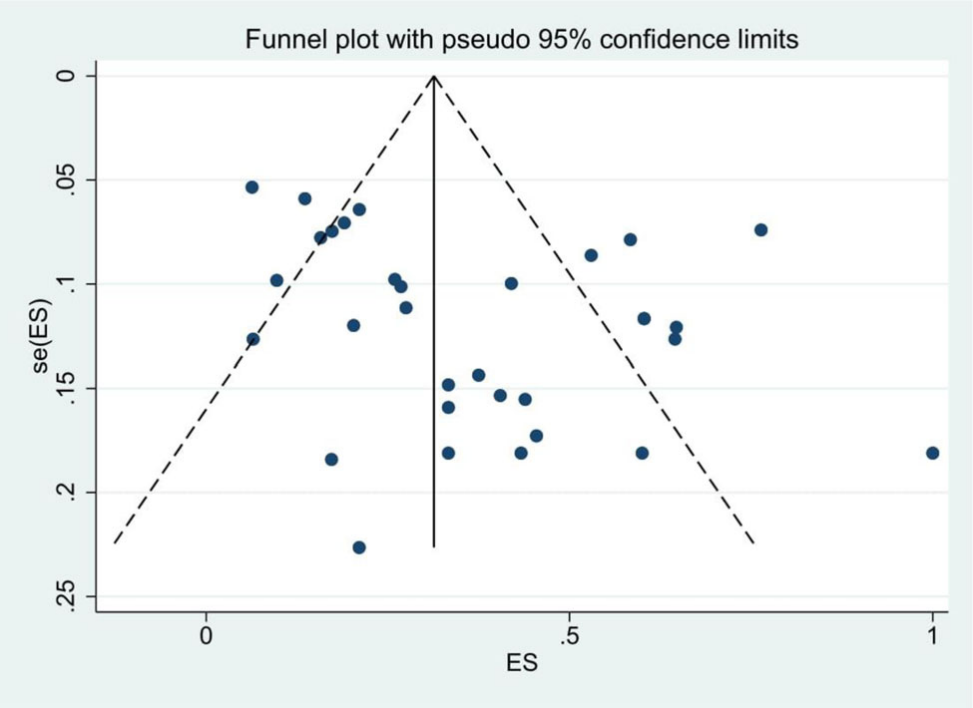
Funnel plot to detect publication bias‐based depression. Circles show selected studies, and the area of each circle is equivalent to the weight of each study. The horizontal axis represents accuracy, and the vertical axis represents the standardized effect.

This study showed that the publication bias was significant among the studies (*p* < .001). Sensitivity test also showed that none of the studies alone had an impressive effect on the overall prevalence of depression and anxiety (Figure 4).

## 4 DISCUSSION

To the extent of our knowledge, this study is the first systematic review and meta‐analysis that investigated the prevalence of overall anxiety and depression in MG people. Thirty‐eight studies were included in this study and the total population was 4108 individuals. We estimated the proportions of depression among MG patients at 36%, (95% CI 28% to 45%), which is higher compared to other autoimmune diseases like multiple sclerosis (30.5%) (Boeschoten et al., 2017) and systemic lupus erythematosus (35.0%) (Moustafa et al., 2020). We also estimated the pooled prevalence of anxiety at 33%, (95% CI 25% to 42%) which is also higher compared to multiple sclerosis (22.1%) (Boeschoten et al., 2017) and systemic lupus erythematosus (25.8%) (Moustafa et al., 2020). According to the finding, the rate of depression is higher than other neurological diseases such as dementia (30.3%) (Orgeta et al., 2015), ALS (34%) (Heidari et al., 2021), multiple sclerosis (30.5%) (Moustafa et al., 2020), and mild cognitive impairment (32%) (Ismail et al., 2017), and elderly patients with hip fracture (23%) (Irvani et al., 2020). Also, rate of depression was lower than in Alzheimer's patients (42%) (Zhao et al., 2016).

MG mainly affects acetylcholine receptors and disturbs their mechanism (O'Connell et al., 2020). In most MG cases, the first symptoms are the weakness of extraocular muscles and ocular misalignment, which is considered as ocular MG. Within 3 years, in around 90% of cases, ocular MG develops to generalized MG (Yang et al., 2016), which is defined by symptoms of weakness and fatigue in skeletal muscles and its effects on swallowing, chewing, breathing, and talking of MG patients (Chu et al., 2019). Disabilities resulting from MG often affect patients’ quality of life and interfere with their daily routine and activity (Vitturi et al., 2021). Additionally, Instability of MG symptoms, prolonged illness, and treatment with steroids can cause psychiatric symptoms in MG patients (Yamamoto et al., 2019). Mood disorders are very common among patients with neurological conditions and MG patients are no exception. It was reported that 41% of MG patients experience mood disorders (Law et al., 2020). According to studies, depressive and anxiety symptoms are very prevalent in MG patients (Cherukupally et al., 2020; Law et al., 2020). In a cross‐sectional study by Alanazy, it was reported that about a quarter of MG patients (26.0%) experienced moderate–severe depression (Alanazy, 2019). In another study by Gavrilov et al., the prevalence of moderate–severe depression among MG patients was estimated at 20.5%. The results of a study by Braz et al. showed that around half (43.75%) of the MG patients had anxiety and 27.5% had depressive symptoms (Braz et al., 2018).

In a literature review by Law et al., it was reported that factors such as older age and disease duration can affect depression and anxiety in MG patients (Law et al., 2020), but our meta‐regression test showed no significant relation between prevalence of anxiety and depression with age and disease duration. Further research is recommended on this matter.

In our study, range of depression varied from 1% to 76% and in anxiety ranged from 3% to 71%. This might be due to the difference in methodological approach and the use of different screening tools. The most used screening tools for anxiety were BAI and HADS and for depression were BDI and HADS. The BAI is one of the most common screening tools for measuring anxiety (Bardhoshi et al., 2016). It was first developed to measure anxiety independently from depression (Toledano‐Toledano et al., 2020). BAI is a self‐report tool focusing on the physical symptoms of anxiety. It contains 21 items to measure the severity of anxiety symptoms and asks patients to rate each symptom on a four‐point scale (0 to 3). Final score can be varied from 0 to 63. Higher scores indicate more severe anxiety symptoms (Julian, 2011; Lee et al., 2018). Studies reported that the BAI is a valid and reliable instrument for assessing anxiety symptoms (Bardhoshi et al., 2016; Toledano‐Toledano et al., 2020). The HADS is a self‐report questionnaire, which was originally developed to assess depression and anxiety in non‐psychiatric patients. The HADS consisted of two sub‐scales: anxiety (HADS‐A) and depression (HADS‐D). This questionnaire has 14 items, 7 items for assessing anxiety and 7 items for assessing depression. The HADS asks individuals to rate each question on a four‐point scale (0 to 3) and the total score can range from 0 to 42, or 0 to 21 for each HADS‐A and HADS‐D (Annunziata et al., 2020; Djukanovic et al., 2017; Julian, 2011; Smarr & Keefer, 2011). Many studies with different populations reported HADS as a reliable and valid instrument to measure anxiety and depression (Bjelland et al., 2002; Bocéréan & Dupret, 2014; Cassiani‐Miranda et al., 2022; Djukanovic et al., 2017). The BDI is a 21 item self‐report questionnaire used to measure depression symptoms and severity. It is probably the most used screening tool for assessing depression in both psychiatric and non‐psychiatric populations (Moher et al., 2009; Крейс et al., 2020). The BDI has several versions including BDI‐I, BDI‐IA, BDI‐II, and BDI‐FS, which unlike others has seven items (Moher et al., 2009). The BDI consisted of 21 items to measure depression symptoms and severity, it asks patients to rate each symptom on a four‐point scale (from 0 to 3), and the final score can range between 0 and 63; higher scores indicate more severe depression symptoms. BDI‐II is a newer version designed to meet the DSM‐IV criteria for depressive disorders and includes items that measure cognitive, emotional, and physical symptoms (García‐Batista et al., 2018; Smarr & Keefer, 2011). Many studies with different types of population considered the BDI as a reliable and valid screening tool (García‐Batista et al., 2018; Lee et al., 2017; Sacco et al., 2016; Smarr & Keefer, 2011). Most of the screening tools for depression and anxiety consider somatic symptoms as an item; it can cause false results for the diagnosis of depression and anxiety in disease or conditions like MG that have somatic symptoms similar to somatic symptoms of depression and anxiety (e.g., fatigue, tiredness, etc.).

Prevalence of depression based on BDI and HADS in our results was 52% and 21%, respectively. We think that the reason for this gap is due to the difference and heterogeneity of cut points. Europe (56%) and Asia (28%) had highest and lowest prevalence of depression among continents. For anxiety, American (53%) and Asian (25%) individuals had highest and lowest prevalence in the present study.

This study is the first study that systematically reviewed the prevalence of anxiety and depression in MG patients. The search strategy, paper screening, and data extraction for this study were comprehensive. Also, PRISMA guidelines were considered for conducting study. Of course, there were some limitations in this study. First, included studies were different in design, screening tools, population origin, publication year, and setting which resulted in heterogeneity among studies. Second, some of the included studies used self‐report questionnaires to assess anxiety and depression in MG patients, which can decrease the reliability of the results. Finally, the possibility of publication bias could not be fully ignored.

## 5 CONCLUSION

According to our results, anxiety and depression are major problems among MG patients and the prevalence of both anxiety and depression is high even when compared to other autoimmune diseases. Symptoms such as depression and anxiety in MG patients are often overlooked or delayed in diagnosis, leading to an increase in severity of symptoms and delayed onset of treatment. Therefore, identifying the related factors and developing effective intervention strategies for MG patients are needed.

## AUTHOR CONTRIBUTIONS

Javad Nadali, Mohammad Eghbal Heidari, and Neda Ghavampour: design of study, literature search, identification and selection, data extraction, quality assessment, conducting analysis, writing paper, and critical revision of paper. Mona Maleki Takhtegahi, Farzaneh Beiranvand, and Omid Narimani Charan: literature search, identification and selection, quality assessment, and critical revision of paper. Tina Arabzadeh and Shahin Salarvand: literature search, identification and selection, and critical revision of paper.

## CONFLICT OF INTEREST

The authors declare no conflict of interests.

## Funding information

None

### PEER REVIEW

The peer review history for this article is available at https://publons.com/publon/10.1002/brb3.2840

## TRANSPARENT PEER REVIEW

The peer review history for this article is available at https://publons.com/publon/10.1002/brb3.2840

## Data Availability

The data that support the findings of this study are available from the corresponding author upon reasonable request.
